# Authors' Reply

**DOI:** 10.1371/journal.pmed.0020032

**Published:** 2005-01-25

**Authors:** Sanjeev Krishna, Timothy Planche

**Affiliations:** St. George's Hospital Medical SchoolLondonUnited Kingdom

We are pleased that Dr. Maitland and colleagues consider our data on volume status (intra- and extracellular) of Gabonese children to be important. We did not consider our children with severe malaria to have intravascular volume depletion for the following reasons. When we measured central venous pressures in a proportion of children on admission, there was no evidence of intravascular volume depletion (median [interquartile range] = 6.5 [3–7.5] cm water), and these values did not change significantly over 24 h, suggesting that our severely ill children had adequate filling pressures. Consistent with this observation, our severely ill children improved rapidly when markers of tissue hypoxia (blood lactate concentrations, tachycardia, and tachypnoea) were serially monitored and children were managed with a relatively conservative fluid replacement regimen. Interestingly, extracellular volume was not increased at admission or afterwards either. Capillary leakage, which commonly accompanies hypovolaemia associated with septic shock, was therefore unlikely to be a significant pathophysiological process in these children with malaria. There may be differences in the severe syndromes of malaria seen in different geographical locations, perhaps accounting for the clinical features attributable to compensated hypovolemic shock reported by Maitland and colleagues. Such differences can be assessed using simple and recently calibrated bioelectrical impedance analysis methodology as well as other techniques that monitor intravascular volumes. The design of optimal fluid management regimens for children with severe malaria can thus be informed not only by theoretical considerations, but also by appropriate physiological assessments.

